# Assessment of Racial and Ethnic Disparities in Access to COVID-19 Vaccination Sites in Brooklyn, New York

**DOI:** 10.1001/jamanetworkopen.2021.13937

**Published:** 2021-06-18

**Authors:** Natasha Williams, Haleigh Tutrow, Paulo Pina, Hayley M. Belli, Gbenga Ogedegbe, Antoinette Schoenthaler

**Affiliations:** 1 Department of Population Health, New York University Grossman School of Medicine, New York; 2 Family Health Centers at New York University Langone Health, New York; 3 Institute for Excellence in Health Equity, New York University Grossman School of Medicine, New York

## Abstract

This cross-sectional study examines the number and location of vaccination sites across 18 districts with varying racial and ethnic demographic characteristics in Brooklyn, New York.

## Introduction

Urban areas throughout the United States have been hardest hit by the COVID-19 pandemic, contributing to a major focus on increasing vaccine uptake. Despite ongoing discourse about vaccine hesitancy, vaccine access among underserved populations is underexplored. As of March 10, 2021, the New York City (NYC) Department of Health and Mental Hygiene reported that 1 250 370 residents had received at least 1 dose of a COVID-19 vaccine, including 19% of the non-Hispanic White population compared with a rate of only 9% among those who identified as African American/Black and Hispanic/Latinx.^1^ In this analysis, we review access to COVID-19 vaccination sites in Brooklyn, the most populated borough in NYC, to better understand disparities in vaccination.

## Methods

Vaccination sites across Brooklyn were evaluated relative to population demographic characteristics and the NYC Government Poverty Measure for this cross-sectional study (Table 1). Demographic and financial data were based on the 2014 to 2018 American Community Survey. Race and ethnicity were self-reported. Vaccination site locations were extrapolated from the NYC Vaccine Finder website, and testing and vaccination data were retrieved from the NYC Health COVID-19: Data website. Descriptive statistics on vaccination rates and distance to sites (measured from the center of each zip code) were averaged (for a mean) for each zip code and included in 18 community districts in Brooklyn. All other tabulations, including demographic characteristics, poverty rates, and total number of vaccination sites, were calculated at the district level. To adjust for differences in population and size of the community districts, population density within each community district was divided by the total number of vaccination sites.

**Table 1.** Brooklyn, New York, Demographic Data by Community District^a^

Brooklyn community district	Total population (thousands)	Population density (thousands), persons per sq mile	%							
			Population below the poverty threshold^b^	Non-Hispanic				Hispanic (of any race)	Female	Male
				White	Black	Asian	Other race^c^			
1	161	37	15.5	63.0	4.5	7.0	2.6	22.9	50.4	49.6
2	141	36	11.0	46.4	25.1	9.3	4.6	14.6	53.3	46.7
3	147	53	21.2	26.0	49.5	3.2	2.3	19.0	53.0	47.0
4	130	56	24.8	18.9	18.4	5.1	2.3	55.3	50.4	49.6
5	159	33	28.7	3.7	52.1	4.1	2.2	37.9	54.4	45.6
6	118	34	9.6	64.1	7.2	7.0	5.4	16.3	52.5	47.5
7	144	34	27.9	24.4	2.6	31.3	1.9	39.8	49.8	50.2
8	131	60	20.4	22.3	58.3	3.8	3.6	12.0	54.0	46.0
9	110	62	20.8	22.8	64.3	1.9	2.6	8.4	54.5	45.5
10	123	31	19.0	54.6	2.1	24.4	2.3	16.6	51.5	48.5
11	189	49	22.5	40.2	1.0	40.8	2.1	15.9	50.8	49.2
12	159	53	27.2	71.1	2.1	14.3	2.2	10.3	49.5	50.5
13	114	33	24.4	55.9	10.7	13.0	3.1	17.3	54.0	46.0
14	162	55	21.5	40.8	30.6	10.7	2.9	15.0	53.1	46.9
15	152	34	18.6	65.5	4.1	17.6	2.9	9.9	51.3	48.7
16	117	46	29.4	2.6	72.1	1.4	1.5	22.4	56.6	43.4
17	135	46	19.5	2.7	86.6	1.3	1.8	7.6	55.9	44.1
18	208	23	14.6	22.7	61.9	5.0	1.8	8.6	54.2	45.8

^a^ Statistical analyses were performed utilizing unrounded values. Community District Demographic Data retrieved from the NYC Planning Population FactFinder. Data updated as of February 17, 2021.

^b^ The poverty threshold is based on the New York City Government Poverty Measure.

^c^ Other races included American Indian/Alaska Native and Native Hawaiian/Other Pacific Islander.

This study was conducted using publicly available data and, as such, did not require institutional review board approval or informed patient consent, in accordance with 45 CFR §46. This study has been compiled using the Strengthening the Reporting of Observational Studies in Epidemiology (STROBE) reporting guideline. Data analysis was performed using R statistical software version 3.6.3 (R Project for Statistical Computing) from February to March 2021.

## Results

This study was implemented in Brooklyn, New York; among 2 604 747 residents, 1 346 395 (51.7%) identified as Latinx or Black and 1 368 835 (52.6%) were female;^2^ the median age was 35.1 years. At the time of data extraction, we identified 87 COVID-19 vaccination sites operating throughout Brooklyn.^3^

The median (range) number of vaccination sites (4 [0-5]) among districts with less than 40% White (non-Hispanic) race/ethnicity was less than the number of vaccination sites (6 [3-8]) among districts with greater than or equal to 40% White (non-Hispanic) race/ethnicity (Table 2). The median population density per site among districts with lower poverty was 6793.6 persons per square mile per site, compared with a ratio nearly double of 11 263.4 persons per square mile per site among districts with higher poverty. The higher vs lower poverty definition was a median cutoff: the 9 community districts with the highest poverty rates vs the 9 community districts with the lowest poverty rates (as defined by the NYC Government Poverty Measure). Of note, district 16 had the highest percentage of the population below the poverty threshold (29.4%) and has 0 vaccination sites.

**Table 2.** Brooklyn, New York, COVID-19 Vaccination Data by Community District^a^^,^^b^

Brooklyn community district	Total sites in district	Nearest site (SD), miles	Sites within 1 mile (SD)	% (SD)		
				Positive tests	Fully vaccinated	Partially vaccinated
1	7	0.3 (0.2)	5.0 (1.7)	6.2 (2.6)	2.7 (0.7)	4.1 (0.6)
2	8	0.4 (0.2)	7.3 (3.0)	3.9 (1.6)	4.7 (1.7)	6.1 (1.5)
3	4	0.3 (0.2)	5.3 (2.0)	6.5 (1.8)	2.7 (1.1)	4.3 (1.1)
4	5	0.3 (0.2)	5.5 (3.1)	8.2 (0.9)	2.2 (0.3)	3.7 (0.3)
5	4	0.5 (0.1)	2.0 (1.0)	8.3 (3.3)	2.8 (1.4)	4.6 (2.1)
6	3	0.2 (0.1)	7.0 (3.2)	2.9 (0.5)	5.5 (1.0)	6.9 (0.9)
7	2	0.3 (0.2)	3.5 (1.7)	7.2 (3.0)	4.0 (1.7)	6.2 (1.4)
8^c^	4	0.4 (0.3)	5.0 (1.2)	6.2 (1.5)	3.2 (1.1)	4.5 (1.1)
9^c^	9	0.4 (0.3)	5.0 (1.2)	6.2 (1.5)	3.2 (1.1)	4.5 (1.1)
10	6	0.4 (0.2)	4.7 (1.5)	8.4 (1.5)	4.3 (1.7)	6.9 (1.7)
11	6	0.4 (0.2)	3.8 (1.7)	10.7 (1.9)	3.4 (0.7)	5.9 (0.7)
12	3	0.3 (0.1)	4.2 (0.8)	10.0 (3.3)	3.8 (0.6)	5.4 (0.9)
13	5	0.4 (0.2)	3.6 (1.1)	12.6 (1.5)	3.9 (1.4)	5.6 (1.0)
14	6	0.3 (0.1)	5.3 (1.9)	9.2 (3.8)	4.0 (0.6)	5.4 (1.1)
15	5	0.5 (0.2)	4.7 (0.6)	13.1 (1.1)	4.4 (1.7)	6.4 (0.8)
16	0	0.7 (0.4)	3.0 (1.4)	7.8 (0.6)	2.2 (0.4)	3.4 (0.3)
17^d^	4	0.3 (NA)	3.0 (NA)	6.2 (NA)	4.1 (NA)	5.2 (NA)
18	10	0.4 (0.2)	4.0 (1.4)	6.3 (0.8)	3.9 (0.9)	5.2 (1.6)

Abbreviation: NA, not available.

^a^ One district with 10 vaccination sites was removed as an outlier when analyzing race/ethnicity because the poverty threshold was the lowest among all 9 districts with race/ethnicity less than 40% White (non-Hispanic). A threshold of 40% White (non-Hispanic) was selected because it was determined to be the numerical midpoint for dividing the 18 Community Districts into 2 equal groups for comparison.

^b^ COVID-19 Vaccination Site Data retrieved from the NYC COVID-19 Vaccine Finder website. COVID-19 Testing and Vaccination Data retrieved from the NYC Health COVID-19 Data page. Data updated as of February 17, 2021.

^c^ Brooklyn Community Districts 8 and 9 comprise the same zip codes, and therefore have the same data set.

^d^ Brooklyn Community District 17 comprises only one zip code, therefore standard deviations do not apply,

## Discussion

The findings of this study suggest that there are substantial vaccination access deserts (areas without ample vaccination resources relative to surrounding areas).^4^ With many preliminary barriers to vaccination uptake, including supply issues, scheduling multiple doses, and a delay for scheduling appointments,^5^ vaccine rollout has been slow. Furthermore, early COVID-19 vaccination efforts in NYC have been focused primarily in White, middle-to-upper class neighborhoods, with the greatest access occurring in these areas.

The analysis presented is generally suggestive of disparities in vaccination site access. This study also had some limitations. The data on race/ethnicity and poverty were dichotomized and analyzed at the community level, and thus do not fully capture socioeconomic status.

Despite the limitations, this study makes a substantial contribution to the literature on vaccine uptake and underserved populations. Patterns of access and inequities, driven by structural determinants, have long existed in Black and Latinx and low-income communities. Without concrete, multilevel solutions, disparities in hospitalizations and deaths due to COVID-19^6^ are likely to continue even while COVID-19 positivity rates continue to trend downward.^1^
